# Widespread Bat White-Nose Syndrome Fungus, Northeastern China

**DOI:** 10.3201/eid2201.151314

**Published:** 2016-01

**Authors:** Joseph R. Hoyt, Keping Sun, Katy L. Parise, Guanjun Lu, Kate E. Langwig, Tinglei Jiang, Shubao Yang, Winifred F. Frick, A. Marm Kilpatrick, Jeffrey T. Foster, Jiang Feng

**Affiliations:** University of California, Santa Cruz, California, USA (J.R. Hoyt, K.E. Langwig, W.F. Frick, A.M. Kilpatrick);; Northeast Normal University, Changchun, China (K. Sun, G. Lu, T. Jiang, J. Feng);; Northern Arizona University, Flagstaff, Arizona, USA (K.L. Parise, J.T. Foster);; Changchun Normal University, Changchun (G. Lu);; Jilin Agricultural University, Changchun (S. Yang)

**Keywords:** white-nose syndrome, *Pseudogymnoascus destructans*, species distribution, Geomyces, bats, fungal disease, Asia, fungi, China

**To the Editor:** Emerging infectious diseases have caused catastrophic declines in wildlife populations, and the introductions of many pathogen have been linked to increases in global trade and travel (*1*). Mapping the distribution of pathogens is necessary to identify species and populations at risk and identify sources of pathogen spillover and introduction. Once pathogen distributions are known, management actions can be taken to reduce the risk for future global spread (*2*).

Bats with symptoms of white-nose syndrome (WNS) were first detected in the United States in 2006, and the disease has subsequently caused precipitous declines in temperate bat populations across eastern North America (*3*,*4*). *Pseudogymnoascus destructans*, the causative agent of WNS, is a cold-growing fungus that infects bats’ skin during hibernation, leading to more frequent arousals from torpor and death (*3*). *P. destructans* is widespread throughout Europe (*5*), but, to our knowledge, its presence in Asia has not been documented.

We sampled bats and hibernacula surfaces (cave walls and ceilings) across northeastern China during 2 visits (June–July 2014 and March 2015) using a previously described swab-sampling technique (*6*). Bats were captured inside caves and at their entrances. DNA was extracted from samples by using a modified QIAGEN DNeasy blood and tissue kit (QIAGEN, Valencia, CA, USA) and tested in duplicate for the presence of *P. destructans* with a quantitative real-time PCR (qPCR) (*6*,*7*).

In the summer of 2014 and winter of 2015, we collected 385 samples from hibernacula surfaces at 12 sites in 3 provinces and 1 municipality (Figure, panel A) and 215 samples from 9 species of bats at 10 sites (summer: *Rhinolophus ferrumequinum, Rhinolophus pusillus, Myotis adversus, Myotis macrodactylus, Myotis pilosus, Myotis chinensis, Murina usseriensis*; winter: *R. ferrumequinum, Murina leucogaster, Myotis petax*). During the summer, *P. destructans* was widely distributed across the study region with positive samples (determined on the basis of qPCR results) obtained from cave surfaces at 9 of 12 sites and from bats at 2 of the 9 sites where bats were sampled (Figure, panel A).

**Figure:**
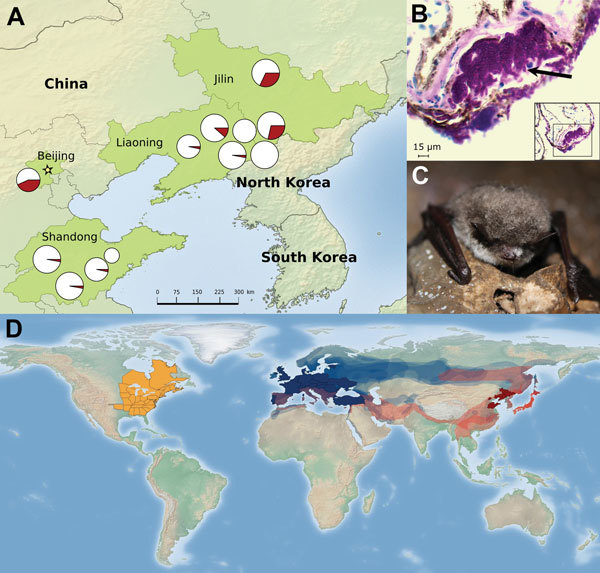
A) Distribution of *Pseudogymnoascus destructans* in cave environments during summer at 9 sites in northeastern China. Pie charts show the prevalence (red indicates fraction of positive samples) of *P. destructans,* and the size of pie graphs indicates the number of samples taken at each site (range 10–35). B) Histologic wing cross-section from *Myotis petax* bat collected in March 2015 with cup-like lesion (arrow) diagnostic of white-nose syndrome (periodic acid–Schiff staining). C) *M. petax* bat in a cave in Jilin, China, showing visible signs of white-nose syndrome, March 2015. D) Documented global distribution of *P. destructans*. Areas in solid dark red and blue represent the provinces and countries in China and Europe, respectively, where *P. destructans* was detected in this study and from previous research (*5*). Semitransparent red and blue regions show the species ranges (range data taken from http://www.iucnredlist.org/) for the bat species detected with *P. destructans* in Asia (n = 6) and Europe (n = 13) (*8*) and possible distribution of *P. destructans*. The solid orange region in North America shows the extent of *P. destructans* spread as of May 15, 2015 (https://www.whitenosesyndrome.org/resources/map).

Prevalence of *P. destructans* was low during summer in the environment (mean prevalence across sites 0.06 ± 0.03) and in bats. Bats of 3 species tested positive for *P. destructans* in the summer: *M. macrodactylus* (1/10), *M. chinensis* (1/1), and *M. ussuriensis* (1/1). *P. destructans* was not detected in bats of 4 other species, of which >20 individual animals of each species were sampled (*R. ferrumequinum*, *R. pusillus*, *M. pilosus*, and *M. adversus*). The low prevalence of *P. destructans* in bats and on hibernacula surfaces in China during the summer was similar to comparable results from studies in North America (*6*). 

In winter, prevalence at the 2 sites we revisited was much higher; 75% of 85 samples from 3 species tested positive, including samples from 16/17 *M. petax* bats. We also detected *P. destructans* in bats from 2 additional species (*R. ferrumequinum* [11/19 bats] and *M. leucogaster* [11/16 bats]).

In addition, during March 2015, we observed visual evidence of *P. destructans* in bats (*M. petax*; Figure, panel C) and obtained 2 fungal cultures from swab specimens taken from these bats. To isolate *P. destructans* from these samples, we plated swab specimens from visibly infected bats on Sabouraud dextrose agar at 10**°**C. We identified potential *P. destructans* isolates on the basis of morphologic characteristics. DNA was then extracted from 2 suspected fungal cultures and tested for *P. destructans* by qPCR, as previously described. 

To further confirm the presence of *P. destructans*, we prepared the fungal isolates for Sanger sequencing (Technical Appendix). The 600-nt amplification products from these 2 isolates were sequenced and found to be 100% identical to the *P. destructans* rRNA gene region targeted for amplification. In addition, using BLAST (http://www.ncbi.nlm.nih.gov/blast.cgi), we found that sequences were a 100% match with isolates from Europe (GenBank accession no. GQ489024) and North America (GenBank accession no. EU884924). This result confirms that the same species of fungus occurs on all 3 continents. We also obtained wing biopsy punches from these bats and found lesions characteristic of WNS by histopathologic examination (Figure, panel B; Technical Appendix). 

The occurrence of *P. destructans* at most sites sampled indicates that this pathogen is widespread in eastern Asia (Figure, panel A). The presence of *P. destructans* in bats from 6 species in China and on bats in 13 species in Europe (*8*) confirms the generalist nature of this fungus and suggests that it may occur throughout Eurasia (Figure, panel D).

Decontamination and restrictions on the use of equipment that has been used in caves in Asia would help reduce the probability of introducing *P. destructans* to uninfected bat populations (e.g., western North America, New Zealand, southern Australia, and temperate areas of South America). These measures would also reduce the risk of introducing new strains of *P. destructans* to regions where bats are already infected (e.g., eastern North America and Europe). These measures are necessary to prevent the devastating effects this pathogen has had on bats in North America and would help maintain the ecosystem services that bats provide (*9**,**10*).

Technical Appendix The Technical Appendix describes the DNA sequencing of fungal isolates to confirm the presence of *Pseudogymnoascus destructans* and histologic examination of bat fungal lesions.
