# Supplementary material for: Widespread Bat White-Nose Syndrome Fungus, Northeastern China
Source: Emerg Infect Dis. 2016 Jan;22(1):140–2. doi: 10.3201/eid2201.151314 (PMC4698868; doi:10.3201/eid2201.151314)
Supplement: Technical Appendix — The Technical Appendix describes the DNA sequencing of fungal isolates to confirm the presence of Pseudogymnoascus destructans and histologic examination of bat fungal lesions. [file 15-1314-Techapp-s1.pdf]

Article DOI: http://dx.doi.org/10.3201/eid2201.151314

# Widespread *Pseudogymnoascus destructans*, Northeastern China

## Technical Appendix

### DNA Amplification and Sequencing

We used conventional PCR with *Pseudogymnoascus*-specific primers developed by Lorch et al. (*1*) [nu-SSI(1506)-184–9-Gd (5′-GGGGACGTCCTAAAGCCT-3′) nu-5.8S-144–3-Gd (5′-TTGTAATGACGCTCGGAC-3′)] for amplifying a fragment of the *P. destructans* ribosomal RNA gene between the small subunit (SSU; 18S) and the internal transcribed spacer region. PCR was conducted by using *Taq* DNA polymerase (Life Technologies, Grand Island, NY, USA), per the manufacturer's instructions. Reactions included 2 μL of extracted DNA (1 ng/μL) in a volume of 20 μL, and the PCR cycling conditions consisted of an initial denaturation at 95°C for 10 min, followed by 40 cycles of 94°C for 1 min, 60°C for 30 s, and 72°C for 1 min, and a final extension of 72°C for 10 min. Five microliters of PCR product were loaded onto a 2% agarose gel and run at 100 V for 45 min. The generation of a 600-nucleotide fragment confirmed the presence of *P. destructans* DNA in the isolates. PCR products were cleaned by using ExoSAP-IT (Affymetrix, Cleveland, OH, USA), following the manufacturer's instructions, and then sequenced by using the PCR primers with BigDye Terminator v3.1 chemistry (Applied Biosystems, Foster City, CA, USA). Reaction products were analyzed on an Applied Biosystems 3130xl automated genetic analyzer (Life Technologies). Sequencing reaction results for complementary strands were assembled and edited in Sequencher 5.3 (Gene Codes, Ann Arbor, MI, USA). These sequences were aligned to the *P. destructans* designated type isolate 20631–21 (GenBank accession no. FJ231098) in BioEdit v7.1.3.0 (*2*) and found to be identical to the aligned region.

## Histology Sample Collection

Two bats that exhibited visible signs of fungal growth were hand captured alive in the field and brought to back to the College of Animal Science and Technology in Changchun, China. An ultraviolet flashlight (395 nm; LEDwholesalers, Hayward, CA, USA) was used to target areas of the wing with potential fungal lesions and a small 3-mm biopsy punch (Miltex, Plainsboro, NJ, USA) specimen was collected (*3*). The wing biopsy specimens were immediately placed in 10% neutral buffered formalin. Wing membranes were rolled to maximize surface area being examined. Protocols described in Meteyer et al. (*4*) were followed for staining, fixing, and sectioning of wing tissue. Stained wing tissues were examined for characteristic lesions of white-nose syndrome at 60× magnification power. Bats were allowed to recover for several days in an isolated flight cage and then returned to the cave where they were captured.

### References

1. Lorch JM, Gargas A, Meteyer CU, Berlowski-Zier BM, Green DE, Shearn-Bochsler V, et al. Rapid polymerase chain reaction diagnosis of white-nose syndrome in bats. J Vet Diagn Invest. 2010;22:224–30. PubMed http://dx.doi.org/10.1177/104063871002200208

2. Hall TA. BioEdit: a user-friendly biological sequence alignment editor and analysis program for Windows 95/98/NT. Nucleic acids symposium series; 1999. p. 95–8.

3. Turner GG, Meteyer CU, Barton H, Gumbs JF, Reeder DM, Overton B, et al. Nonlethal screening of bat-wing skin with the use of ultraviolet fluorescence to detect lesions indicative of white-nose syndrome. J Wildl Dis. 2014;50:566–73. PubMed http://dx.doi.org/10.7589/2014-03-058

4. Meteyer CU, Buckles EL, Blehert DS, Hicks AC, Green DE, Shearn-Bochsler V, et al. Histopathologic criteria to confirm white-nose syndrome in bats. J Vet Diagn Invest. 2009;21:411–4. PubMed http://dx.doi.org/10.1177/104063870902100401
